# Targeted Doxorubicin-Loaded Dendronized Gold Nanoparticles

**DOI:** 10.3390/pharmaceutics15082103

**Published:** 2023-08-09

**Authors:** Lance T. Dockery, Marie-Christine Daniel

**Affiliations:** Department of Chemistry and Biochemistry, University of Maryland Baltimore County (UMBC), Baltimore, MD 21250, USA; lado1@umbc.edu

**Keywords:** dendrons, gold nanoparticles, doxorubicin, targeted delivery, prostate cancer

## Abstract

Dendronized nanoparticles, also called nanoparticle-cored dendrimers, combine the advantages of nanoparticles and dendrimers. These very stable and polyvalent nanoparticles can be used for diverse applications. One such application is drug delivery, because the dendrons can enhance the density of the payload. In this report, we describe the design of multifunctional gold nanoparticles (AuNPs) coated with poly(propylene imine) (PPI) dendrons that contain both prostate cancer active targeting and chemotherapeutic drugs. The PPI dendron is a good candidate for the design of drug delivery vehicles because of its ability to induce a proton sponge effect that will enhance lysosomal escape and intracellular therapeutic delivery. The chemotherapeutic drug used is doxorubicin (DOX), and it was linked to the dendron through a hydrazone acid-sensitive bond. Subsequent acidification of the AuNP system to a pH of 4–5 resulted in the release of 140 DOX drugs per nanoparticles. In addition, the PPI dendron was conjugated via “click” chemistry to an EphA2-targeting antibody fragment that has been shown to target prostate cancer cells. In vitro cell viability assays revealed an IC50 of 0.9 nM for the targeted DOX-bearing AuNPs after 48 h incubation with PC3 cells. These results are very promising upon optimization of the system.

## 1. Introduction

Nanocarriers enable the delivery of therapeutic drugs with improved target specificity through a combination of prolonged circulation, active targeting, and payload conjugation chemistry [1,2]. The prolonged circulation of nanoparticles can be tuned with careful attention to size and surface coating; for instance, by using polymers, such as polyethylene glycol (PEG) or other polymers, thereby improving nanoparticle pharmacokinetics and biodistribution [3]. The incorporation of active targeting ligands (e.g., peptides, antibodies, etc.) and the conjugation of cytotoxic therapeutic drugs via stimuli-responsive attachments can further enhance nanocarrier target specificity [4].

The pharmaceutical industry has developed many different nanocarriers that have either already been approved by the Food and Drug Administration (FDA) or are currently undergoing clinical trials for the treatment of cancer and other diseases. Lipid nanoparticles have been approved for the delivery of cytotoxic cancer therapeutics (e.g., Doxil) and have in recent years emerged as an efficient method to deliver nucleic acid therapies, like the vaccines against COVID-19 developed by Pfizer and Moderna [5,6]. Various other polymer-based nanoparticles include polymer–protein conjugates, polymer–small molecule conjugates, dendrimers, and polymer nanoparticles [7,8]. Some recently approved polymer conjugate therapeutics include Jivi (Bayer, hemophilia A) and Palynziq (Biogen, phenylketonuria) [9]. Several dendrimer-based therapeutics are currently undergoing clinical trials for cancer, such as AZD0466 (Starpharma/AstraZeneca, leukemia) and DEP ^®^ cabazitaxel (Starpharma, prostate cancer) [10].

The predominance of polymer conjugates and dendrimers in FDA drug approvals and clinical trials highlights an active area of research and a key focus of the nanomedicine community [11]. Dendrimers are highly branched spherical and symmetrical structures composed of a polymer backbone (e.g., poly(amidoamine), PAMAM and poly(propylene imine), PPI) that grows outward from a core into well-defined generations [12,13,14,15]. The well-defined and monodisperse nature of dendrimers allows for the attachment of multivalent molecules, such as therapeutic drugs, active targeting moieties, and imaging agents [16,17,18]. The synergy of gold nanoparticles (AuNP) with dendrimers or dendrons through either ligand adsorption or covalent–coordinate bonding to gold has led to promising dendritic AuNP hybrids with tunable properties [19], including the potential for efficient drug delivery [20,21,22,23]. The ability to densely pack drug-loaded dendritic structures around AuNPs with well-defined sizes can dramatically improve therapeutic efficacy over dendrimers on their own [24,25,26].

Gold nanoparticles are a promising nanomedicine platform because they can be prepared in monodisperse and well-defined sizes and are relatively inert once coated with biocompatible ligands [27,28,29]. The strong coordinate–covalent interaction between gold nanoparticles and suitable sulfur-containing ligands enables the formation of very stable nanoparticles (NPs). This allows for different thiolated or disulfide ligands to be mixed and imparts multifunctionality (e.g., therapeutic, targeting, imaging) onto the nanoparticle surface. Additionally, AuNPs inherently possess the ability to enhance ultrasound and radiotherapies and to act as contrast agents with X-ray imaging modalities [30,31].

The combination of dendrons (non-spherical asymmetric branched molecules) with AuNPs has been of particular interest to our group. The coating of AuNPs with dendrons offers several advantages. First, the use of dendrons as ligands increases the stability of the resulting gold nanoparticles as well as the density of the functionalities around them. Dendronized AuNPs are stable in biological media towards lyophilization and with respect to shelf life [21,32,33]. Second, adding dendrons onto NPs leads to the formation of giant dendrimers (nanoparticle-cored dendrimers) in one step [21]. Third, the synthesis of dendronized NPs allows for the use of a mixture of dendrons with diverse termini, giving rise to multifunctional nanoconstructs in a single step. Finally, the multifunctionality of the NPs can be easily tuned by varying the ratios of dendrons used to coat them. Dendrons may be synthesized in well-controlled reactions with high yields from commercially available starting materials to give molecules with varied terminal group functionalities and a disulfide (thioctic acid, TA) focal point for attachment to AuNPs [34,35,36]. We have previously reported the synthesis of poly(propylene imine) (PPI) dendrons having different charges and their incorporation onto the surface of AuNPs to form negatively charged, zwitterionic, and positively charged dendronized AuNPs [21]. These dendronized AuNPs had robust aqueous and serum stability, could withstand several rounds of lyophilization, and showed very low in vitro cytotoxicity. We have also shown that the PPI dendron could be conjugated to doxorubicin (DOX) as a model chemotherapeutic via an acid-labile hydrazone bond at a ratio of three DOX/dendron, giving PPI-DOX [37].

Doxorubicin is one of the most effective antitumor drugs against solid tumors [38], but its clinical usage has been limited due to its low bioavailability and severe side effects, such as cardiotoxicity [39]. Its main mode of action involves intercalation within DNA base pairs resulting in bent DNA strands, which leads to inhibition of both DNA and RNA synthesis, generating the formation of reactive oxygen species and oxidative stress [40].

Herein, we report the preparation of multifunctional dendronized AuNPs possessing acid-labile conjugated DOX drugs and active targeting moieties (Fab) conjugated through copper-free click chemistry. A PPI-N_3_ dendron was synthesized and then mixed with PPI-DOX dendrons during a ligand exchange step on AuNPs using different ratios of DOX/N_3_. The multifunctional AuNPs reported here were made possible with a novel ligand exchange method starting from PPI-CO_2_H-dendronized AuNPs and using a large excess of new dendrons rather than the traditional ligand exchange starting from citrate-coated AuNPs. The stabilizing PPI-CO_2_H dendron mixed with PPI-DOX and PPI-N_3_ produced multifunctional AuNPs bearing DOX and azide functionality with a good size and polydispersity index (PDI). The increase in size and change in surface charge, as measured by dynamic light scattering (DLS), could be used to monitor the ligand exchange and subsequent bioconjugation to the targeting ligand (Fab). The multifunctional drug-loaded AuNP-DOX-N_3_ showed rapid acid-triggered DOX release at a pH of 4.5. The targeted nature of AuNP-DOX/Fab was observed in vitro using transmission electron microscopy (TEM). Finally, in vitro cytotoxicity assays revealed an IC_50_ against PC3 cells of 0.9 nM for AuNP-DOX/Fab.

Although AuNPs have previously been reported for the delivery of DOX [41,42,43], this is the first study reporting the use of dendronized AuNPs bearing both acid-labile DOX and a Fab ligand targeting prostate tumors.

## 2. Materials and Methods

### 2.1. General Methods

Solvents and reagents were obtained from commercial sources (e.g., Sigma Aldrich, St. Louis, MO, USA; Fischer Scientific, Pittsburg, PA, USA; VWR international, Radnor, PA, USA; TCI Chemicals, Portland, OR, USA; etc.) and used without additional purification unless specified otherwise. Glassware, stir bars, and other containers used for gold nanoparticle synthesis and ligand exchange reactions were cleaned with freshly prepared aqua regia and then washed thoroughly (6×) with 18 mΩ ultrapure water from the Milli-Q water purification system. Gold chloride (HAuCl_4_) and trisodium citrate (Na_3_C_6_H_5_O_7_) were obtained from Electron Microscopy Sciences (Hatfield, PA, USA) and used without purification. Dendrons used for gold nanoparticle ligand exchange reactions were prepared as described previously [21,34]. Gold nanoparticle centrifugation was performed using either benchtop (Thermo Fisher Sorvall Legend Micro 21R, Waltham, MA, USA) or an Avanti J-E Centrifuge (Beckman Coulter, Brea, CA, USA) and a JA-20 rotor. Gold nanoparticles were purified using either regenerated cellulose dialysis membranes (MWCO 12K-14K Da) obtained from Fisher Scientific (Pittsburg, PA, USA) or with successive rounds of centrifugation and filtration, as described below.

UV-Vis absorbance data were obtained from a Beckman Coulter DU 730 UV-Vis spectrophotometer. Fluorescence measurements were collected using a Horiba Jobin Yvon FluoroMax-3 instrument using the manufacturer′s DataMax software (version 32). Unless otherwise specified, excitation wavelength for doxorubicin was set at 470 nm. Dynamic light scattering (DLS) and zeta potential data were collected from a Zetasizer Nano-ZS system (Malvern Instruments, Southborough, MA, USA) using a 633 He-Ne laser and operating on backscatter mode at an angle of 173°. The software used to analyze the data was Zetasizer version number 7.11 by Malvern Instruments.

Next, 1× PBS (pH 7.4) was prepared using volumetric glassware and ultrapure water (resistivity of 18 MΩ·cm). The 1× PBS was filtered through Cytiva Whatman ™ Puradisc 0.2 μm syringe filters prior to use with nanoparticles or diluted to 0.1× PBS (pH 7.4) using ultrapure water. Solutions of NaCl and saturated sodium bicarbonate (Fisher Scientific) were prepared using the corresponding salts and then filtered through Cytiva Whatman ™ Puradisc 0.2 μm syringe filters before use with nanoparticles.

PC-3 prostate cancer cells were provided by Dr. Charles Bieberich (Department of Biology, University of Maryland, Baltimore County). They were grown in Falcon-brand cell culture flasks using RPMI media supplemented with FBS and penstrep (Gibco, Thermo Fisher Scientific, Waltham, MA, USA) at 37 °C in a humidified atmosphere of 5% CO_2_. Cytotoxicity studies involving AuNP-CO_2_H, AuNP-DOX, AuNP-Fab, and AuNP-DOX/Fab were conducted with PC-3 cells using MTT reagent (Thermo Fisher Scientific) according to the manufacturer′s recommended protocol. These studies are described in detail below.

### 2.2. Synthesis of PPI-N_3_ Dendron

PPI-N_3_ (2)—A solution of TA-TEG-G2NH_2_ [21] (27 mg, 0.03 mmol) was dissolved in 4 mL anhydrous MeOH and stirred in a round-bottom flask. To this stirring solution was added an azide-modified Michael acceptor [44] (121.7 mg, 0.4469 mmol) and LiBr (35 mg, 0.40 mmol). The reaction was stirred vigorously at rt. for 48 h. After 48 h, the reaction was concentrated under reduced pressure to give a yellow oil, which was purified by LH20 size exclusion chromatography in MeOH. The pure product was obtained as a yellow oil (62 mg, 65%). The purity of the product was confirmed by ^1^H NMR (400 MHz, D20) δ 7.15 (br, 2H), 6.87 (br, 2H), 4.08 (br, 2H), 3.81 (br, 2H), 3.62–3.32 (m, 77H), 3.25–2.10 (m, 63H), and 2.07–0.51 (m, 76H), and FT-IR spectroscopy ν = 3380 cm^−1^, 2921.3 cm^−1^, 2851.9 cm^−1^, 2095 cm^−1^ (N_3_), 1732.7 cm^−1^, 1462 cm^−1^, 1246.4 cm^−1^, and 1119 cm^−1^.

### 2.3. Synthesis of Dendronized Gold Nanoparticles

AuNP-Citrate (3)—A gold chloride stock solution was prepared by dissolving HAuCl_4_ (101.7 mg) in 100 mL Millipore ultrapure water in a volumetric flask to give a concentration of 2.99 mM. Separately, 100 mL of a 5% *w*/*v* tri-sodium citrate stock solution was prepared using 5 g sodium citrate and Millipore ultrapure water in a volumetric flask. The 2.99 mM 100 mL HAuCl_4_ stock solution was transferred to a large two-neck round-bottom flask and diluted to a concentration of 0.3 mM using Millipore ultrapure water (~1 L). This solution was stirred and heated to 90 °C. Once the solution reached 90 °C, 14.06 mL of the sodium citrate stock solution (molar ratio 1:6 HAuCl_4_:Na_3_citrate) was added to the reaction in one addition, and the reaction was brought to reflux for 1 h. After 1 h, the reaction was cooled to rt overnight before it was analyzed by UV-Vis and DLS to characterize the formed AuNPs. The particle size (DLS by number) was observed to be 13.6 nm (PDI = 0.067).

AuNP-CO_2_H (4)—A solution of AuNP-citrate (269 mL, 6 nM) was stirred in a freshly cleaned glass round-bottom flask. Separately, 37.7 mg PPI-CO_2_H [21] (**1**) dendron (25.6 μmol) was dissolved in 1 mL Millipore ultrapure water at a pH of 8 (basified with NaHCO_3_) and then filtered through a syringe filter (GE Life Sciences, 0.22 μm) to give the dendron solution to be added to the AuNP-citrate solution. An excess of PPI-CO_2_H dendron was added such that 20× the total theoretical ligand coating of the AuNP surface area was in the solution. The AuNP-citrate solution was first basified to a pH of 8 (monitored with pH strips) using 1 mL of filtered saturated sodium bicarbonate solution. Then, the previously prepared PPI-CO_2_H dendron solution was added to the stirring basic AuNP-citrate solution at rt. The reaction color immediately turned to a darker red. After addition of the dendron solution, an additional 5 mL filtered saturated sodium bicarbonate solution was added to the stirring AuNPs to keep the pH at 8. The reaction was then stirred overnight at rt for a total of 15 h. After 15 h, a 1 mL aliquot was centrifuged twice (15,000× *g*, 45 min, 4 °C), resuspending in between with Millipore ultrapure water with a pH of 8, and examined via DLS, UV-Vis, and zeta potential, which indicated completion of the reaction. This solution of AuNP-CO_2_H was stored without purification until further use. The DLS size by number was 16.8 nm, and the zeta potential was −42.0 mV.

AuNP- Fab (6)—A solution of AuNP-CO_2_H was first centrifuged 1× (9k× *g*, 45 min, 4 °C), the supernatant was removed, and the AuNPs were resuspended in water with a pH of 8 with 0.02% TWEEN20 and 25% DMSO. The solution of AuNP-CO_2_H (18 mL, 6.0 nM) was stirred in a 20 mL scintillation vial that had been freshly cleaned with aqua regia. To this stirring solution was added PPI-CO_2_H (8 × molar excess) and PPI-N_3_ (2 × molar excess) from stock solutions in water with a pH of 8 and DMSO, respectively. The total excess of dendron was added such that 10× the total theoretical ligand coating of the AuNP surface area was in the solution. The AuNPs were stirred for 17 h at room temperature and then centrifuged 1× (11k× *g*, 1 h, 10 °C) and 2× (9k× *g*, 45 min, 4 °C), removing the supernatant each time and resuspending the particles in 1× PBS (pH 8) with 0.02% TWEEN20. The washed AuNP-N_3_ solution (**5**) was then stirred and Fab-DBCO was added from a stock solution in 1× PBS (est. 20 Fab/AuNP), and the reaction was stirred at room temperature for 1 h. After 1 h, the AuNP-Fab solution was centrifuged 2× (9k× *g*, 45 min, 4 °C). The AuNPs were characterized by DLS, zeta potential, and UV-Vis spectroscopy. The solution was stored in 1× PBS (pH 8) with 0.02% TWEEN20 in the refrigerator as a stock solution until it was used for TEM experiments. TWEEN20 was removed by 3 rounds of centrifugation and resuspension in 1× PBS prior to in vitro cytotoxicity experiments.

AuNP-DOX—A solution of AuNP-CO_2_H (30 mL, 6 nM) was first centrifuged 1× (9k× *g*, 45 min, 4 °C). Then, the supernatant was removed, and the AuNPs were resuspended in pH 8 water with 0.02% TWEEN20 and 25% DMSO. The solution of AuNP-CO_2_H (10 mL, 6 nM) was stirred in a 20 mL scintillation vial that had been freshly cleaned with aqua regia. To this stirring solution was added a combined solution (at a pH of 8) of 1.56 mL (1 mg/mL stock) PPI-CO_2_H (5 × molar excess) and 1.28 mL (2.51 mg/mL stock) PPI-DOX (5 × molar excess) from stock solutions in pH 8 water and DMSO, respectively. The total excess of dendron was added such that 10x the total theoretical ligand coating of the AuNP surface area was in the solution. The AuNPs were stirred for 22 h at room temperature and then centrifuged 1× (11k× *g*, 1 h, 10 °C) and 2× (9k× *g*, 45 min, 4 °C), removing the supernatant each time and resuspending the particles in 1× PBS (pH 8) with 0.02% TWEEN20. The resulting AuNP-DOX solution was then used for stimuli-responsive acid release experiments.

AuNP-DOX/Fab (8)—A solution of AuNP-CO_2_H (10 mL, 6 nM) was first centrifuged 1× (9k× *g*, 45 min, 4 °C). The supernatant was removed, and the AuNP pellet was resuspended in pH 8 water with 0.02% TWEEN20 and 25% DMSO. The resulting solution of AuNP-CO_2_H (10 mL, 6 nM) was stirred in a 20 mL scintillation vial that had been freshly cleaned with aqua regia. To this stirring solution was added 2 mg PPI+N_3_ (10 × molar excess, from 11.7 mg/mL stock solution in DMSO) and 0.5 mg PPI-DOX (2.5 × molar excess, from 2.51 mg/mL stock solution in DMSO). The total excess of dendron was added such that 12.5× the total theoretical ligand coating of the AuNP surface area was in the solution. The AuNPs were stirred for 22 h at room temperature and then centrifuged 1× (11k× *g*, 1 h, 10 °C) and 2× (9k× *g*, 45 min, 4 °C), removing the supernatant each time and resuspending the particles in 1× PBS (pH 8) with 0.02% TWEEN20. The washed AuNP-DOX/N_3_ solution (**7**) was then stirred, Fab-DBCO was added from a stock solution in 1× PBS (est. 20 Fab/AuNP), and the reaction was stirred at room temperature for 1 h. After 1 h, the AuNP-DOX/Fab solution was centrifuged 2× (9k× *g*, 45 min, 4 °C), and the pellet was resuspended in 1× PBS (pH 8) with 0.02% TWEEN20 and characterized by DLS, zeta potential, and UV-Vis spectroscopy. The solution was stored in 1× PBS (pH 8) with 0.02% TWEEN20 in the refrigerator until further experiments. TWEEN20 was removed by 3 rounds of centrifugation and resuspension in 1× PBS prior to in vitro cytotoxicity experiments.

### 2.4. Stimuli-Responsive DOX Release from AuNPs

A solution of AuNP-DOX (1 mL, 83 pM) was centrifuged and resuspended in 1 mL of 0.1× PBS. The solution was acidified to a pH of 4.5 through the addition of 1M HCl, as monitored by pH test strips. A separate control solution of AuNP-DOX/N_3_ (1 mL, 83 pM) in 1× PBS at a pH of 7.4 was also prepared. The solution of acidified solution with a pH of 4.5 was allowed to sit at room temperature in the dark until measuring the fluorescence of the solution (1 h to 19 days). The AuNPs solution was thoroughly mixed using a vortexer prior to each measurement. The AuNP solutions at a pH of 4.5 and a pH of 7.4 were monitored using fluorescence at 15 min, 30 min, 1.5 h, 3 h, 5 h, and 19 days. Rapid release of DOX from AuNP-DOX/N_3_ was observed within 5 h at a pH of 4.5. No increase in fluorescence of the AuNP-DOX/N_3_ at a pH of 7.4 was observed. 

In another experiment, a solution of 1 mL AuNP-DOX was also centrifuged, resuspended in 1 mL of 0.1× PBS, and acidified to a pH of 4.5. However, it was then transferred to a slide-a-lyzer cassette (10,000 MWCO) and dialyzed against 0.1 M PBS at a pH of 4.5. Fluorescence spectra of the dialysate were recorded at 1 h, 2 h, 3 h, 4 h, 5 h, 6 h, 7 h, 24 h, and 48 h. The DOX release trend observed was similar to the release directly measured from the AuNP-DOX solution (see the previous paragraph).

### 2.5. In Vitro AuNP Experiments

PC3 prostate cancer cells were seeded in a 96-well plate in RPMI media at a density of 10,000 cells/well. After 24 h at 37 °C in a humidified atmosphere of 5% CO_2_, AuNPs were added to the wells at the specified concentrations. All experiments were repeated in triplicates. The cells were incubated for 24, 48, and 72 h at 37 °C in a humidified atmosphere of 5% CO_2_. At 24, 48, or 72 h time points, 15 μL MTT solution (5 mg/mL in 1× PBS) was added to the cells and incubated for 4 h. After 4 h, the medium was removed, and the formazan crystals were dissolved in 200 μL 1× PBS with sodium dodecyl sulfate (SDS, 5% *v*/*v*). The 96-well plate was read after 3 h on an automated plate reader.

## 3. Results and Discussion

### 3.1. Design and Synthesis of AuNP-Fab Bioconjugates

**Figure 1:**
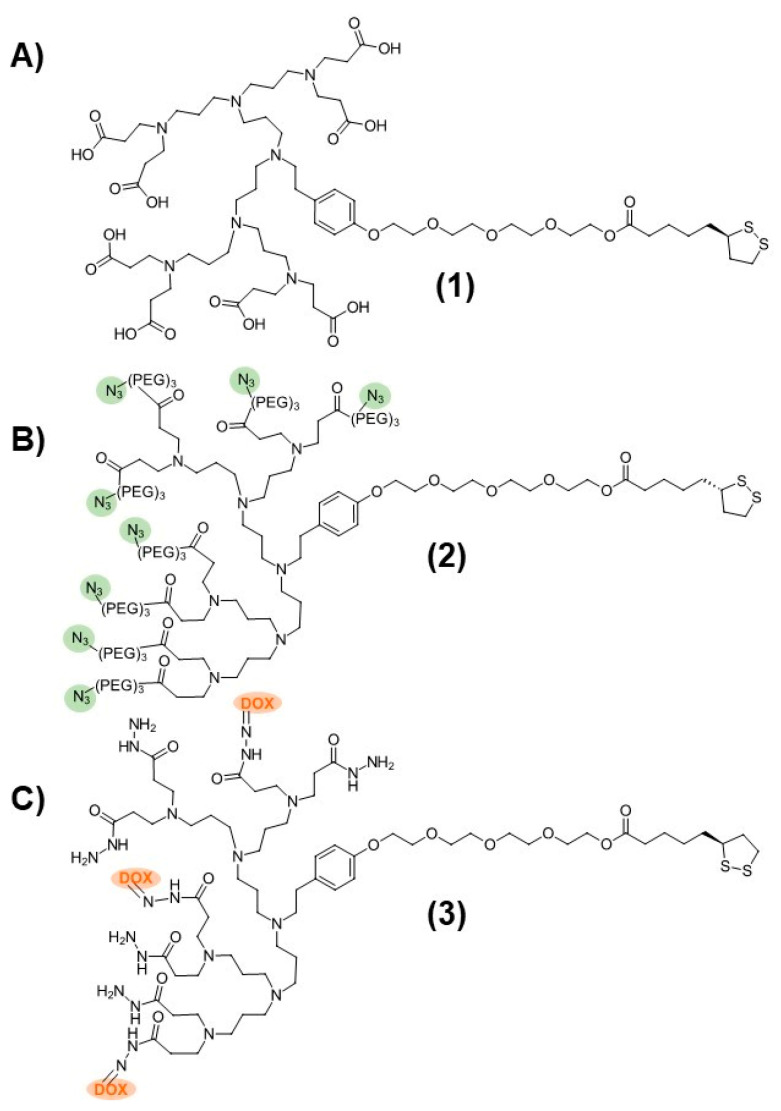
Structures of (**A**) PPI-CO_2_H (**1**), (**B**) PPI-N_3_ (**2**), and (**C**) PPI-DOX (**3**) poly(propylene imine) dendrons.

The dendronized AuNP-Fab system contained two dendron components that were prepared separately: a carboxyl-terminated stabilizing dendron (PPI-CO_2_H **1**, Figure 1) and an azide-functionalized PPI dendron (PPI-N_3_ **2**, Figure 1) capable of bioconjugation to suitable targeting ligands. The third-generation PPI-CO_2_H dendron **1** was prepared as described previously starting from TA-TEG-G2NH_2_ and methyl acrylate, followed by aqueous hydrolysis of the ester groups and dialysis [21]. The synthesis of the PPI-N_3_ dendron **2** was carried out in a similar manner. Starting from TA-TEG-G2NH_2_, a custom-made azide-PEG_3_ Michael acceptor was reacted to give the corresponding PPI-N_3_ dendron **2** with a 65% yield. The PPI-DOX conjugate **3** (Figure 1), bearing three DOX molecules per PPI dendron, was synthesized as described in a previous publication [37].

**Figure 2:**
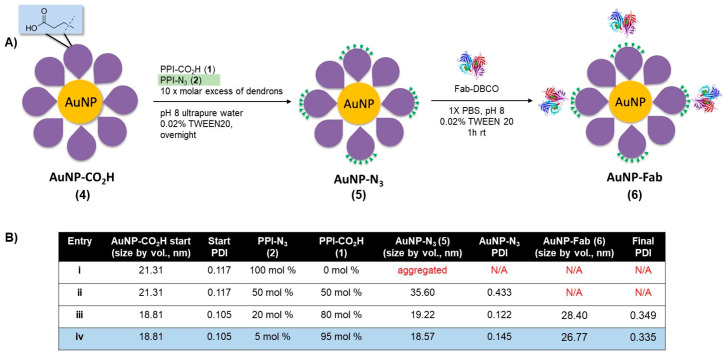
(**A**) Synthesis of AuNP-N_3_ and AuNP-Fab, and (**B**) optimization of ligand exchange ratios for AuNP-N_3_ and AuNP-Fab stability.

As a first attempt to prepare dendronized AuNP-Fab bioconjugates, PPI dendrons **1** and **2** were directly added to a basic solution of AuNP-citrate, as we reported previously, for the preparation of AuNP-CO_2_H [21]. However, this reaction did not proceed well, and rapid aggregation of the AuNPs was observed. We hypothesized that the nonpolar nature of azide PPI dendron **2** relative to dendron **1** (evidenced by low aqueous solubility) contributed to the instability of the resulting dendronized AuNPs in the aqueous solution. To overcome this lack of stability, we developed a novel ligand exchange reaction starting with the more stable AuNP-CO_2_H (**4**, Figure 2) itself prepared from AuNP-citrate. A ten-fold molar excess (compared to the theoretical number of ligands per NP) of various ratios of azide and carboxylate PPI dendrons **1** and **2** was added to the reaction, as shown in Figure 2. The addition of 0.02% TWEEN20 overall favored the formation of stable AuNP-N_3_ (**5**) by limiting the occurrence of aggregation. The overnight ligand exchange reaction proceeded well when using up to 20% PPI-N_3_ dendron, with the size remaining relatively stable at 18 nm (DLS size by volume) and a PDI < 0.15 as measured by DLS. Ratios of between 5% and 20% PPI-N_3_ dendrons gave comparable results. However, greater ratios of PPI-N_3_ dendron (50–100%) led to some aggregation. The use of 50% PPI-N_3_ resulted in partial aggregation, reflected by a significant increase in size (from 21 to 36 nm) and in PDI (from 0.122 to 0.433). And the AuNPs subjected to ligand exchange with 100% PPI-N_3_ completely aggregated. The resulting stable AuNP-N_3_ (**5**) were purified by centrifugation and washing to remove excess unreacted dendrons and were then conjugated to the targeting ligand. The size of AuNP-N_3_ using 20% PPI-N3 dendron increased only slightly compared to AuNP-CO_2_H (from 18.8 nm to 19.2 nm), which is in agreement with the small size difference between PPI-CO_2_H and PPI-N_3_ (PPI-N_3_ contains an extra tetraethylene glycol spacer). Also, the zeta potential of AuNP-N_3_ is less negative than AuNP-CO_2_H (−15.2 mV versus −41.8 mV), reflecting the replacement of some negatively charged PPI-CO_2_H dendrons with positively charged PPI-N_3_ dendrons (protonated in water). 

Bioconjugation of AuNP-N_3_ (**5**) to an EphA2-targeting antibody fragment (Fab) derivatized with a clickable DBCO group occurred in 1× PBS with 0.02% TWEEN20 at room temperature over the course of 1 h. AuNP-N_3_ solutions with 5% and 20% azide content (Figure 2, entries iii and iv) were reacted with excess Fab-DBCO (20 Fab/AuNP), and the progress of the reaction was monitored by an increase in size by DLS. Both 5% and 20% azide content AuNP-N_3_ properly coupled with Fab-DBCO (size ~ 4–5 nm) based on an increase in AuNP size of 8–10 nm for the resulting nanobioconjugate **6** (Figure 2). AuNP-Fab resulting from 5% azide content (Figure 2B, entry iv) displayed the lowest PDI and was less likely to show some aggregation during purification (two to three rounds of successive centrifugation and washing), so this protocol was identified as the optimal bioconjugation to give AuNP-Fab 6.

### 3.2. Design and Synthesis of Multifunctional AuNP-DOX/Fab

**Figure 3:**
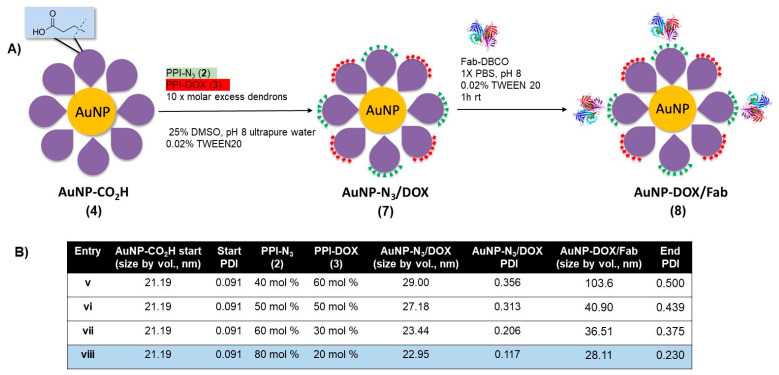
(**A**) Synthesis of doxorubicin- and azide-functionalized AuNP-N_3_/DOX, and (**B**) optimization of ligand exchange ratios for AuNP-N_3_/DOX and for AuNP-DOX/Fab stability.

With the protocol to prepare AuNP-Fab (**6**) established, a similar approach was taken to synthesize DOX-loaded AuNP-DOX/Fab. Given the limited polar nature of the PPI-DOX dendron, different conditions were required to achieve efficient ligand exchange and subsequent bioconjugation (Figure 3). As with **6**, the synthesis of AuNP- DOX/N_3_ (**7**) required starting with AuNP-CO_2_H (**4**), and the ligand exchange with PPI-DOX (**3**) and PPI-N_3_ (**2**) dendrons was carried out overnight in a 25% DMSO aqueous solution using 10× molar excess dendrons (compared to the total theoretical number of ligands per NP) to give AuNP-DOX/N_3_ (**7**). For this reaction, the best conditions used 80% PPI-N_3_ and 20% PPI-DOX (Figure 3, Entry viii) and produced stable nanoparticles with low PDI (0.117), even after two to three rounds of successive centrifugation and washing. After the last round of purification, the AuNP-DOX/N_3_ was resuspended in 1× PBS with 0.02% TWEEN20 for targeting ligand conjugation.

The targeting ligand conjugation of AuNP-N_3_/DOX (**7**) to Fab-DBCO was carried out as described with AuNP-N_3_ (**5**) using an excess of Fab (20 Fab/AuNP, Figure 3, Entries v–viii). All formulations of AuNP- DOX/N_3_ properly coupled with Fab-DBCO, as evidenced by an increase of 5–10 nm in the AuNP-DOX/Fab product, as measured by DLS. However, only entry viii, using 80% PPI-N_3_ and 20% PPI-DOX, gave particles with a relatively low PDI (0.230) that were stable during purification by two to three rounds of successive centrifugation and washing. With AuNP-Fab and AuNP-DOX/Fab prepared, a full characterization of the DOX-loaded AuNPs was carried out, and an evaluation of the drug release as well as of the in vitro targeting properties was undertaken.

The reproducibility of the nanoconjugate preparations was assessed by DLS: over six independent experiments, with same ligand ratios, were carried out and showed consistent size increases and PDI values.

### 3.3. Characterization of Multifunctional AuNP-DOX/Fab

**Figure 4:**
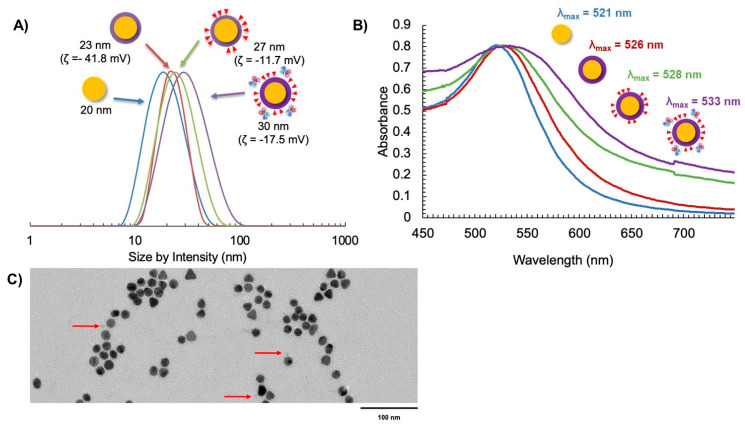
Characterization of AuNP synthesis using (**A**) DLS and zeta potential, (**B**) UV-Vis spectroscopy, and (**C**) TEM with uranyl acetate negative staining (the red arrows indicate the presence of Fab fragments on the surface of AuNPs).

Following the ligand exchange with the PPI-N_3_/PPI-DOX dendrons and bioconjugation to the targeting Fab, the final AuNP-DOX/Fab particles were characterized by DLS (size by intensity), zeta potential, UV-vis spectroscopy and transmission electron microscopy (TEM) (Figure 4). The size of the initial citrate-capped AuNPs was observed to be 20 nm (Figure 4A). Ligand exchange with PPI-CO_2_H resulted in a small increase in size to 23 nm and a strongly negative zeta potential (−41.8 mV). The 3 nm size increase corresponds to the replacement of citrate with the PPI-CO_2_H dendrons. The zeta potential stays negative (AuNP-citrate is also negatively charged with typical zeta potential between −40 and −50 mV) because PPI-CO_2_H dendrons predominantly exist as PPI-CO_2_^-^ in water. Further ligand exchange with PPI-N_3_/PPI-DOX dendrons resulted in another small increase in the size of the AuNPs to 27 nm. This additional 4 nm size increase can have two explanations: first, the addition of DOX to the dendron increases its size; second, the lower polarity of DOX could lead to the expansion of the previously tight packing of the PPI-CO_2_H dendrons at the AuNP surface (Figure 4A). The zeta potential of AuNP-N_3_/DOX was also shifted to a less negative value (−11.7 mV), reflecting the decrease in anionic CO_2_^-^ groups in favor of the more positively charged PPI-N_3_ and PPI-DOX dendrons on the AuNP. These positive charges come from the protonation of the azide groups and of the primary amine of DOX in water, respectively. The subsequent bioconjugation to give AuNP-DOX/Fab further increased the AuNP size to 30 nm, and the zeta potential shifted to a slightly more negative value (−17.5 mV), indicating successful conjugation of the targeting Fab, as this ligand has been previously observed to impart a negative charge on nanoparticles (Figure 4A) [45,46].

The AuNP-DOX/Fab synthesis was also supported by UV-Vis spectroscopy, as measured by the surface plasmon resonance (SPR) wavelength of absorption as the AuNP coating changed [47]. It was observed that λ_SPR_ = 521 nm for AuNP-citrate (Figure 4B). This value increased to λ_SPR_ = 528 nm for AuNP-DOX/N_3_ and further increased to λ_SPR_ = 533 nm for AuNP-DOX/Fab (Figure 4B). The broadening of the SPR peak after the addition of DOX is most likely due to the absorption of PPI-DOX (Figure S2) that occurs at a similar wavelength to AuNP but with a much broader peak (from 440 to 600 nm, with a λ_max_ at around 505 nm) [37,48]. The addition of Fab fragments accentuates this broadening, because these 50 kDa proteins are significantly changing the environment of the AuNP core. The AuNP-DOX/Fab coating was also observed using TEM and negative staining with uranyl acetate. While it is difficult to see targeting ligand attachment for all AuNPs due to the possibility that ligands may be above or below the field of view, some Fab fragments could be observed (Figure 4C). 

**Figure 5:**
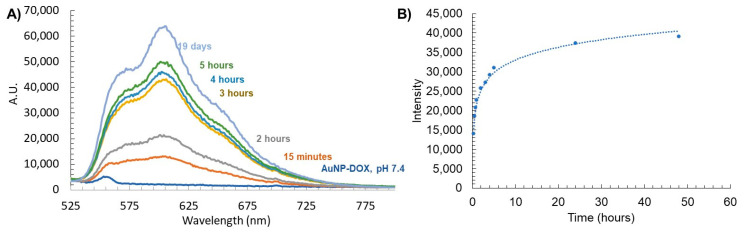
(**A**) Stimuli-responsive release of DOX from AuNP-DOX at a pH of 4.5 measured by fluorescence, and (**B**) fluorescence of free DOX released from AuNPs found in dialysate with a pH of 4.5.

Next, we characterized the stimuli-responsive nature of these particles in response to acidification. The use of the acid-labile acyl hydrazone bond as a linkage of DOX to the dendron leads to acid-sensitive AuNP-DOX that is expected to release DOX at an acidic pH. To evaluate this release, AuNPs containing DOX dendrons were dissolved in 1× PBS at a pH of 7.4 and then acidified to a pH of 4.5. We chose to carry out the drug release study at a pH of 4.5 in order to mimic the lysosomal environment; indeed, lysosomal compartments are known to have a pH of 4.5 to 5 [49]. The release profile of DOX from this acidic AuNP-DOX system was observed by measuring the direct fluorescence of the AuNP solution as well as through dialysis and observation of aliquots of the dialysate over time (Figure 5). At a pH of 7.4, the AuNP-DOX solutions showed no DOX fluorescence signal (Figure 5A). The lack of fluorescence is due to the quenching of DOX on the AuNP surface due to the closely arranged or π-stacked DOX molecules [50]. In addition, the proximity of DOX to the AuNP core is another possible reason for its fluorescence quenching through energy transfer from DOX to the gold core [51,52]. Acidification to a pH of 4.5 caused the DOX–hydrazone bond to be cleaved, and an initial rapid release of DOX was observed at 15 min (Figure 5A). In the dialysis experiment, the release profile of DOX from AuNP-N_3_/DOX obtained by analyzing the dialysate (Figure 5B) matched the release profile using the AuNP-solution-based fluorescence data (Figure 5A). The majority of the DOX cleaved from the AuNPs and found in the dialysate was observed up to 5 h after acidification, and the release curve significantly leveled out after that point. This rapid release profile indicates that the AuNP system may be ideal as a drug delivery platform because rapid release of DOX from AuNPs is necessary during endosome acidification so that it can escape and reach the cytosol and then the nucleus. Indeed, DOX must enter the nucleus and intercalate with DNA to cause cellular cytotoxicity. This release displays similar trends to a recent study using polymeric NPs that encapsulate a pH-sensitive DOX prodrug [53].

This DOX release study also allowed us to estimate the number of DOX released per AuNP. The fluorescence intensity of the dialysate at 24 h was compared to the corresponding calibration curve, and knowing the concentration of the AuNPs, it was concluded that about 140 DOX were released per AuNP within 24 h. 

### 3.4. In Vitro Properties of Multifunctional AuNP-DOX/Fab

**Figure 6:**
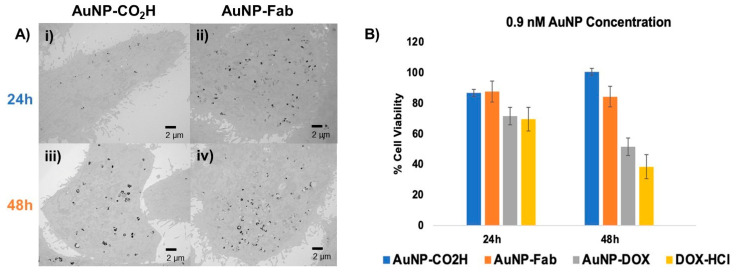
(**A**) In vitro evaluation of Fab-targeted (**ii**,**iv**) AuNPs and untargeted AuNP-CO_2_H (**i**,**iii**) using TEM and (**B**) MTT cytotoxicity assay of PC3 cancer cells incubated for 24–48 h with 0.9 nM untargeted AuNP-CO_2_H (4), targeted AuNP-Fab (6) and targeted, DOX-loaded AuNP-DOX/Fab (9), with 0.9 µM DOX-HCl as a positive cytotoxicity control.

As is usually performed for in vitro studies of targeted drug nanocarriers [54,55], we have compared the cellular uptake as well as the cytotoxicity of our targeted and non-targeted AuNPs. To assess the cellular uptake of targeted AuNPs, TEM was used to monitor PC3 prostate cancer cells treated for 24 and 48 h with 0.6 nM AuNP-CO_2_H or 0.6 nM AuNP-Fab, respectively. After 24 h or 48 h, respectively, the medium with AuNPs was removed, the PC3 cells were washed with 1× PBS, and the fixative was added. The resulting TEM images showed an increased uptake of AuNP-Fab (Figure 6A ii,iv) compared to AuNP-CO_2_H (Figure 6A i,iii) in terms of both the number of AuNPs and the density of the accumulated AuNPs inside the PC3 prostate cancer cells (Figure 6A). This difference is most striking after 24 h, for which the number and density of AuNP-Fab is significantly higher than for AuNP-CO_2_H, indicating that the targeting ligand promoted efficient uptake of AuNPs within the first 24 h.

To further evaluate this AuNP system as a drug delivery platform, an in vitro MTT cytotoxicity assay was performed using AuNP-CO_2_H, AuNP-Fab, AuNP-DOX/Fab, and DOX-HCl (as a positive control). PC3 prostate cancer cells were treated with dendronized AuNPs at concentrations of 0.6 or 0.9 nM, and DOX-HCl as a positive control was added at a concentration of 0.9 µM, roughly equivalent to the total DOX loaded on the AuNP-DOX/Fab, as calculated by the AuNP SPR (Supplementary Materials) [47]. At 24 and 48 h after treatment with AuNPs or DOX-HCl, cell viability was measured with an MTT assay (Figure 6B). At 24 and 48 h at both concentrations, the -CO_2_H and Fab-conjugated control AuNPs (**4/6**) were overall nontoxic to the PC3 prostate cancer cells. However, AuNP-DOX/Fab (**9**) showed a concentration-dependent cytotoxic behavior: while treatment of PC3 cells with 0.6 nM AuNP-DOX/Fab led to lower cytotoxicity (about 70% cell viability after 48 h, Figure S3), the use of 0.9 nM AuNP-DOX/Fab (**9**) gave significantly increased PC3 cell cytotoxicity (about 52% cell viability after 48 h) as compared to AuNPs **4** (about 85% cell viability after 48 h) and **6** (about 94% cell viability after 48 h), and cell death was comparable to the positive DOX control (about 38% cell viability after 48 h) (Figure 6B). This means that the IC_50_ of AuNP-DOX/Fab is around 0.9 nM AuNPs or 0.13 µM DOX (because we found 140 DOX/AuNP). These data also suggest that the control dendronized AuNPs **4** are generally nontoxic to PC3 prostate cancer cells under physiological conditions, while the targeted and drug-loaded AuNP-DOX/Fab shows in vitro potential as a prostate cancer therapeutic. These results are more promising than some obtained by other DOX-loaded NPs, which showed similar potency after 48 h exposure for over 10 times higher concentration than in this study (7 µM DOX in ref. [47] versus 0.13 µM DOX in this work) [52]. In addition, although the antiproliferative activity of AuNP-DOX/Fab is slightly lower than the free DOX one, a main advantage of this nanoparticle formulation is the stimuli-responsive linkage of DOX to the nanoparticle, which keeps DOX as a pro-drug, and thus safe, until it enters the cancer cells. This is a significant advantage over free DOX because this will avoid side effects, especially the cardiotoxicity associated with free DOX.

## 4. Conclusions

In conclusion, we have successfully prepared a targeted stimuli-responsive DOX-loaded dendronized AuNP platform that has potential as a cancer therapeutic. Using ten times lower concentrations of DOX, it has shown similar potency to other NP formulations at treating castration-resistant prostate cancer, with an IC_50_ around 0.9 nM AuNP (or 0.13 µM DOX). This platform was prepared from a novel ligand exchange reaction between negatively charged dendronized AuNP-CO_2_H and azide-functionalized and/or doxorubicin-conjugated PPI dendrons. These dendrons could be added to a solution of AuNP-CO_2_H at varied ratios to give multifunctional AuNP-DOX/N_3_. The AuNPs prepared here were conjugated through azide/DBCO click chemistry to an antibody fragment to allow for nanoparticle active targeting. Doxorubicin was conjugated to the PPI platform using pH-sensitive acyl hydrazone bonds. The resulting AuNPs were observed to have targeted uptake in PC3 prostate cancer cells, as observed by TEM, and selective cytotoxicity of AuNP-Fab/DOX when compared to AuNP-Fab and AuNP-CO_2_H. Overall, these results show that using dendrons on AuNPs can compensate for low ratios of drug-loaded ligands on the NPs (20% DOX ligands on the AuNPs still led to 140 DOX/AuNP) and lead to lower IC_50_ than existing NPs. This opens the door to AuNPs with higher multifunctionality and higher cytotoxicity by combining other drug-loaded dendrons (such as docetaxel or cabazitaxel) with DOX on a single NP using various ratios. This AuNP system may also be expanded to dendrons with MRI contrast agents to afford a powerful theranostic platform.

## Data Availability

The data presented in this study are available upon request from the corresponding author.

## Supplementary Materials

The following supporting information can be downloaded at: https://www.mdpi.com/article/10.3390/pharmaceutics15082103/s1, Calculation for AuNP ligand exchange reaction; Figure S1: ^1^H NMR (D_2_O) of TA-TEG-G3N_3_ (2); Figure S2: UV-Vis spectrum of PPI-DOX dendron; Figure S3: MTT assay using 0.6 nM AuNPs.

## References

[1] Villanueva-Flores F., Castro-Lugo A., Ramírez O.T., Palomares L.A. (2020). Understanding cellular interactions with nanomaterials: Towards a rational design of medical nanodevices. Nanotechnology.

[2] Peer D., Karp J.M., Hong S., FaroKhzad O.C., Margalit R., Langer R. (2007). Nanocarriers as an emerging platform for cancer therapy. Nat. Nanotechnol..

[3] Suk J.S., Xu Q., Kim N., Hanes J., Ensign L.M. (2016). PEGylation as a strategy for improving nanoparticle-based drug and gene delivery. Adv. Drug Deliv. Rev..

[4] Yoo J., Park C., Yi G., Lee D., Koo H. (2019). Active Targeting Strategies Using Biological Ligands for Nanoparticle Drug Delivery Systems. Cancers.

[5] Shan X., Gong X., Li J., Wen J., Li Y., Zhang Z. (2022). Current approaches of nanomedicines in the market and various stage of clinical translation. Acta Pharm. Sin. B.

[6] Rouge J. (2023). RNA and nanocarriers: Next generation drug and delivery platform take center stage. Trends Biotechnol..

[7] Seidi F., Jenjob R., Crespy D. (2018). Designing Smart Polymer Conjugates for Controlled Release of Payloads. Chem. Rev..

[8] Begines B., Ortiz T., Perez-Aranda M., Martinez G., Merinero M., Arguelles-Arias F., Alcudia A. (2020). Polymeric Nanoparticles for Drug Delivery: Recent Developments and Future Prospects. Nanomaterials.

[9] Ekladious I., Colson Y.L., Grinstaff M.W. (2019). Polymer-drug conjugate therapeutics: Advances, insights and prospects. Nat. Rev. Drug Discov..

[10] Wang J., Li B., Qiu L., Qiao X., Yang H. (2022). Dendrimer-based drug delivery systems: History, challenges, and latest developments. J. Biol. Eng..

[11] Thakor P., Bhavana V., Sharma R., Srivastava S., Singh S.B., Mehra N.K. (2020). Polymer–drug conjugates: Recent advances and future perspectives. Drug Discov. Today.

[12] Tomalia D.A. (2005). Birth of a new macromolecular architecture: Dendrimers as quantized building blocks for nanoscale synthetic polymer chemistry. Prog. Polym. Sci..

[13] Esfand R., Tomalia D.A. (2001). Poly(amidoamine) (PAMAM) dendrimers: From biomimicry to drug delivery and biomedical applications. Drug Discov. Today.

[14] Numai S., Yoto R., Kimura M., Simanek E.E., Kitano Y. (2023). Click Chemistry of Melamine Dendrimers: Comparison of “Click-and-Grow” and “Grow-Then-Click” Strategies Using a Divergent Route to Diversity. Molecules.

[15] Lim J., Simanek E.E. (2012). Triazine dendrimers as drug delivery systems: From synthesis to therapy. Adv. Drug Deliv. Rev..

[16] Menjoge A.R., Kannan R.M., Tomalia D.A. (2010). Dendrimer-based drug and imaging conjugates: Design considerations for nanomedical applications. Drug Discov. Today.

[17] Lim J., Guan B., Nham K., Hao G., Sun X., Simanek E.E. (2019). Tumor uptake of triazine dendrimers decorated with four, sixteen, and sixty-four PSMA-targeted ligands: Passive versus active tumor targeting. Biomolecules.

[18] Falanga A., Del Genio V., Kaufman E.A., Zannella C., Franci G., Weck M., Galdiero S. (2021). Engineering of Janus-like dendrimers with peptides derived from glycoproteins of herpes simplex virus type 1: Toward a versatile and novel antiviral platform. Int. J. Mol. Sci..

[19] Elbert K.C., Jishkariani D., Wu Y., Lee J.D., Donnio B., Murray C.B. (2017). Design, Self-Assembly, and Switchable Wettability in Hydrophobic, Hydrophilic, and Janus Dendritic Ligand–Gold Nanoparticle Hybrid Materials. Chem. Mater..

[20] Nabavifard S., Jalili S., Rahmati F., Vasseghian Y., Ali G.A.M., Agarwal S., Gupta V.K. (2020). Application of Dendrimer/Gold Nanoparticles in Cancer Therapy: A Review. J. Inorg. Organomet. Polym. Mater..

[21] Saha Ray A., Ghann W.E., Tsoi P.S., Szychowski B., Dockery L.T., Pak Y.J., Li W., Kane M.A., Swaan P., Daniel M.-C. (2019). Set of Highly Stable Amine- and Carboxylate-Terminated Dendronized Au Nanoparticles with Dense Coating and Nontoxic Mixed-Dendronized Form. Langmuir.

[22] Dockery L., Daniel M.-C. (2018). Dendronized Systems for the Delivery of Chemotherapeutics. Adv. Cancer Res..

[23] Enciso A.E., Doni G., Nifosì R., Palazzesi F., Gonzalez R., Ellsworth A.A., Coffer J.L., Walker A.V., Pavan G.M., Mohamed A.A. (2017). Facile synthesis of stable, water soluble, dendron-coated gold nanoparticles. Nanoscale.

[24] Zhang S., Chen C., Xue C., Chang D., Xu H., Salena B.J., Li Y., Wu Z.-S. (2020). Ribbon of DNA Lattice on Gold Nanoparticles for Selective Drug Delivery to Cancer Cells. Angew. Chem. Int. Ed..

[25] Kalimuthu K., Lubin B.C., Bazylevich A., Gellerman G., Shpilberg O., Luboshits G., Firer M.A. (2018). Gold nanoparticles stabilize peptide-drug-conjugates for sustained targeted drug delivery to cancer cells. J. Nanobiotechnol..

[26] Khutale G.V., Casey A. (2017). Synthesis and characterization of a multifunctional gold-doxorubicin nanoparticle system for pH triggered intracellular anticancer drug release. Eur. J. Pharm. Biopharm..

[27] De Souza C.D., Nogueira B.R., Rostelato M.E.C.M. (2019). Review of the methodologies used in the synthesis gold nanoparticles by chemical reduction. J. Alloys Compd..

[28] Kimling J., Maier M., Okenve B., Kotaidis V., Ballot H., Plech A. (2006). Turkevich method for gold nanoparticle synthesis revisited. J. Phys. Chem. B.

[29] Enustun B.V., Turkevich J. (1963). Coagulation of Colloidal Gold. J. Am. Chem. Soc..

[30] Fu L., Ke H.-T. (2016). Nanomaterials incorporated ultrasound contrast agents for cancer theranostics. Cancer Biol. Med..

[31] Gao Q., Zhang J., Gao J., Zhang Z., Zhu H., Wang D. (2021). Gold Nanoparticles in Cancer Theranostics. Front. Bioeng. Biotechnol..

[32] Love C.S., Ashworth I., Brennan C., Chechik V., Smith D.K. (2006). Dendron-protected Au nanoparticles—Effect of dendritic structure on chemical stability. J. Colloid Interface Sci..

[33] Cho T.J., Zangmeister R.A., MacCuspie R.I., Patri A.K., Hackley V.A. (2011). Newkome-Type Dendron-Stabilized Gold Nanoparticles: Synthesis, Reactivity, and Stability. Chem. Mater..

[34] Pan H., Grow M.E., Wilson O., Daniel M.-C. (2013). A new poly(propylene imine) dendron as potential convenient building-block in the construction of multifunctional systems. Tetrahedron.

[35] Ghann W.E., Aras O., Fleiter T., Daniel M.-C. (2012). Syntheses and Characterization of Lisinopril-Coated Gold Nanoparticles as Highly Stable Targeted CT Contrast Agents in Cardiovascular Diseases. Langmuir.

[36] Daniel M.-C., Grow M.E., Pan H., Bednarek M., Ghann W.E., Zabetakis K., Cornish J. (2011). Gold nanoparticle-cored poly(propyleneimine) dendrimers as a new platform for multifunctional drug delivery systems. N. J. Chem..

[37] Dockery L., Zalesak-Kravec S., Kane M.A., Daniel M.-C. (2022). Modular and efficient synthesis of a poly (propylene imine) (PPI) dendron applied to acid-sensitive doxorubicin conjugation. Tetrahedron.

[38] Ibrahim A.M., Nady S., Shafaa M.W., Khalil M.M. (2022). Radiation and chemotherapy variable response induced by tumor cell hypoxia: Impact of radiation dose, anticancer drug, and type of cancer. Radiat. Environ. Biophys..

[39] Gomari H., Moghadam M.F., Soleimani M., Ghavami M., Khodashenas S. (2019). Targeted delivery of doxorubicin to HER2 positive tumor models. Int. J. Nanomed..

[40] Korga A., Ostrowska M., Iwan M., Herbet M., Dudka J. (2019). Inhibition of glycolysis disrupts cellular antioxidant defense and sensitizes HepG2 cells to doxorubicin treatment. FEBS Open Bio.

[41] Aryal S., Grailer J.J., Pilla S., Steeber D.A., Gong S. (2009). Doxorubicin conjugated gold nanoparticles as water-soluble and pH-responsive anticancer drug nanocarriers. J. Mater. Chem..

[42] Zhang C., Zhang F., Han M., Wang X., Du J., Zhang H., Li W. (2020). Co-delivery of 5-fluorodeoxyuridine and doxorubicin via gold nanoparticle equipped with affibody-DNA hybrid strands for targeted synergistic chemotherapy of HER2 overexpressing breast cancer. Sci. Rep..

[43] Faid A.H., Shouman S.A., Badr Y.A., Sharaky M. (2022). Enhanced cytotoxic effect of doxorubicin conjugated gold nanoparticles on breast cancer model. BMC Chem..

[44] Welser K., Perera M.D.A., Aylott J.W., Chan W.C. (2009). A facile method to clickable sensing polymeric nanoparticles. Chem. Commun..

[45] Florinas S., Liu M., Fleming R., Van Vlerken-Ysla L., Ayriss J., Gilbreth R., Dimasi N., Gao C., Wu H., Xu Z.-Q. (2016). A Nanoparticle Platform to Evaluate Bioconjugation and Receptor-Mediated Cell Uptake Using Cross-Linked Polyion Complex Micelles Bearing Antibody Fragments. Biomacromolecules.

[46] Chen S., Florinas S., Teitgen A., Xu Z.-Q., Gao C., Wu H., Kataoka K., Cabral H., Christie R.J. (2017). Controlled Fab installation onto polymeric micelle nanoparticles for tuned bioactivity. Sci. Technol. Adv. Mater..

[47] Haiss W., Thanh N.T.K., Aveyard J., Fernig D.G. (2007). Determination of size and concentration of gold nanoparticles from UV−Vis spectra. Anal. Chem..

[48] Chen C.G., Nardi A.N., Giustini M., D’Abramo M. (2022). Absorption behavior of doxorubicin hydrochloride in visible region in different environments: A combined experimental and computational study. Phys. Chem. Chem. Phys..

[49] Chen R., Jäättelä M., Liu B. (2020). Lysosome as a Central Hub for Rewiring PH Homeostasis in Tumors. Cancers.

[50] Sherlock S.P., Tabakman S.M., Xie L., Dai H. (2011). Photothermally Enhanced Drug Delivery by Ultrasmall Multifunctional FeCo/Graphitic Shell Nanocrystals. ACS Nano.

[51] Wang F., Wang Y.-C., Dou S., Xiong M.-H., Sun T.-M., Wang J. (2011). Doxorubicin-Tethered Responsive Gold Nanoparticles Facilitate Intracellular Drug Delivery for Overcoming Multidrug Resistance in Cancer Cells. ACS Nano.

[52] Nizamov T.R., Garanina A.S., Grebennikov I.S., Zhironkina O.A., Strelkova O.S., Alieva I.B., Kireev I.I., Abakumov M.A., Savchenko A.G., Majouga A.G. (2018). Effect of Iron Oxide Nanoparticle Shape on Doxorubicin Drug Delivery Toward LNCaP and PC-3 Cell Lines. Bionanoscience.

[53] Czupiel P., Delplace V., Shoichet M. (2020). Nanoparticle delivery of a pH-sensitive prodrug of doxorubicin and a mitochondrial targeting VES-H8R8 synergistically kill multi-drug resistant breast cancer cells. Sci. Rep..

[54] Muhamad N., Plengsuriyakarn T., Na-Bangchang K. (2018). Application of active targeting nanoparticle delivery system for chemotherapeutic drugs and traditional/herbal medicines in cancer therapy: A systematic review. Int. J. Nanomed..

[55] Trabulo S., Aires A., Aicher A., Heeschen C., Cortajarena A.L. (2017). Multifunctionalized iron oxide nanoparticles for selective targeting of pancreatic cancer cells. Biochim. Biophys. Acta Gen. Subj..

