# Supplementary material for: Targeted Doxorubicin-Loaded Dendronized Gold Nanoparticles
Source: Pharmaceutics. 2023 Aug 9;15(8):2103. doi: 10.3390/pharmaceutics15082103 (PMC10459818; doi:10.3390/pharmaceutics15082103)
Supplement: Supplementary file 1 [file pharmaceutics-15-02103-s001.zip › pharmaceutics-2469146-supplementary.pdf]

Supplementary Materials

Targeted Doxorubicin-loaded Dendronized Gold Nanoparticles

Lance, T. Dockery†, Marie-Christine Daniel†

† Department of Chemistry and Biochemistry, University of Maryland Baltimore County (UMBC), Baltimore, Maryland 21250, United States

Calculation for AuNP ligand exchange reaction:

Size of AuNPs by number was measured by DLS to be 12 nm in this calculation.

Calculation of AuNPs concentration

Absorbance of AuNP-citrate at 450 nm was measured by UV-Vis to be 0.674.

Following reported calculations[1] , and using 1.09×10^8 as the extinction coefficient at 450 nm for 12 nm AuNP:

$(0.674 / 1.09 \times 10^8) * 0.500L = 3.09 \times 10^{-9}$ moles AuNP

Calculation of ligand coating

Ligand coating of AuNP calculated from NP surface area divided by the dendron area on a AuNP ($0.4\ nm^2$)[2]:

$(4 \times \pi \times r^2) / 0.4 = 1131$ ligands per AuNP

Calculation of dendrons for ligand exchange

(3.09×10^{-9} moles AuNP) × (mol weight of dendron) × (1131 ligands) × (mol excess) = grams of dendron to be added.

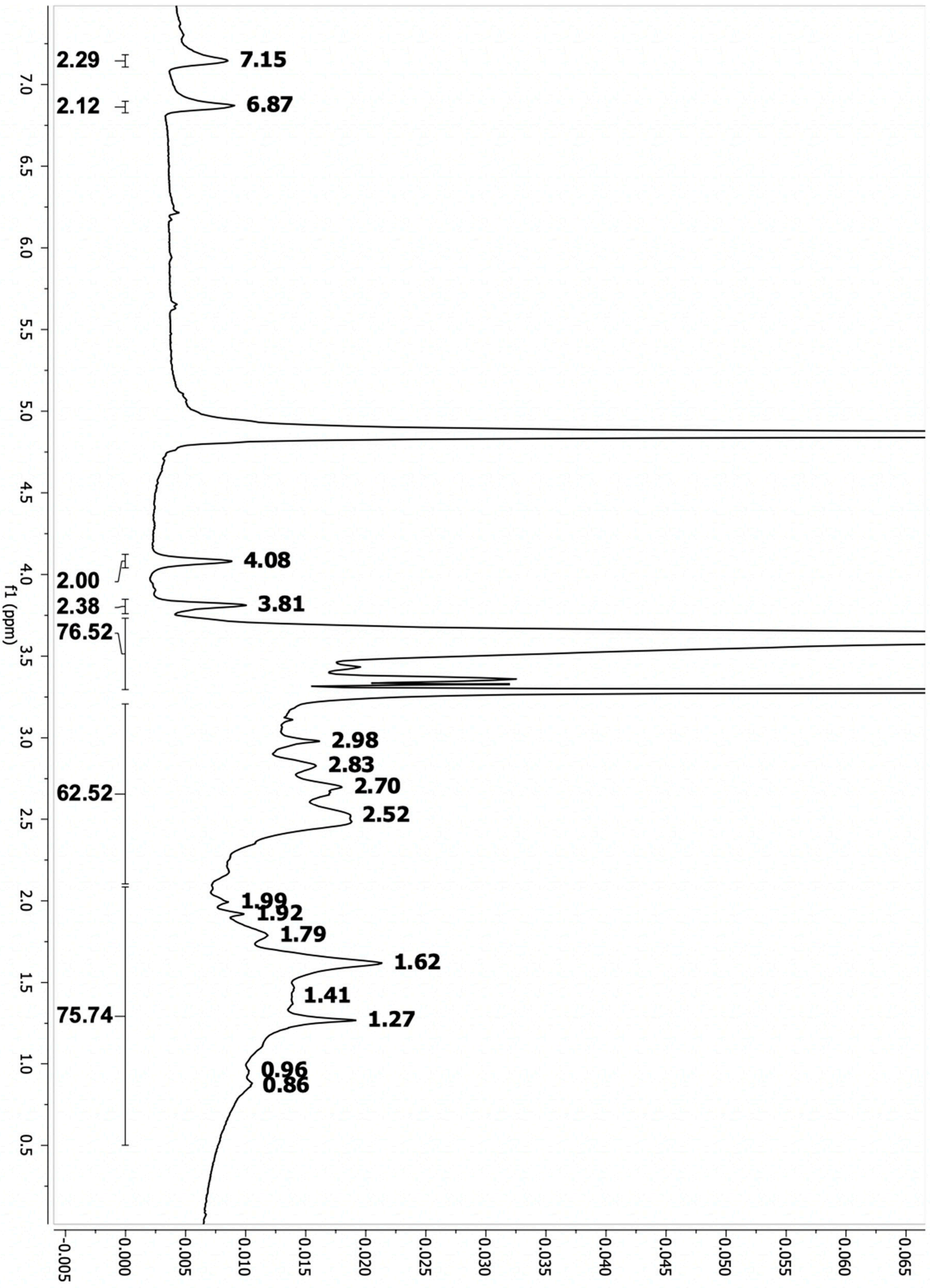
7.15
6.87
4.08
3.81
2.98
2.83
2.70
2.52
1.99
1.92
1.79
1.62
1.41
1.27
0.96
0.86
2.29
2.12
2.00
2.38
76.52
62.52
75.74
f1 (ppm)

Figure S1. ^{1}H NMR (D_2O) of TA-TEG-G3N_3 (**2**).

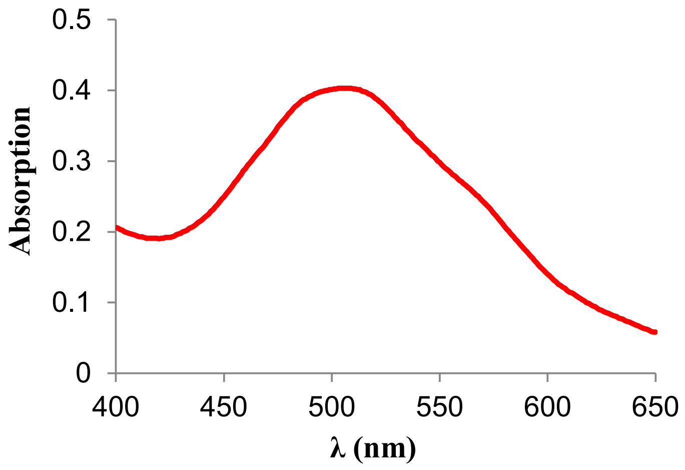

Figure S2. UV-Vis absorption spectrum of PPI-DOX in water

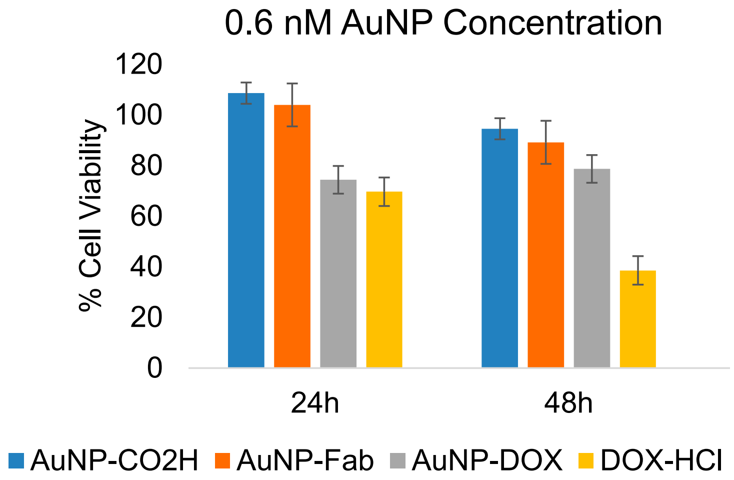

Figure S3. MTT cytotoxicity assay of PC3 cancer cells incubated for 24-48 hours with 0.6 nM untargeted AuNP-CO_2H (**4**), targeted AuNP-Fab (**6**) and targeted, DOX-loaded AuNP-DOX/Fab (**9**), with 0.6 μM DOX-HCl as a positive cytotoxicity control.

References

(1) Haiss, W.; Thanh, N. T. K.; Aveyard, J.; Fernig, D. G. Determination of size and concentration of gold nanoparticles from UV-Vis spectra. *Analytical Chemistry* **2007**, *79* (11), 4215-4221. DOI: 10.1021/ac0702084.
(2) Saha Ray, A.; Ghann, W. E.; Tsoi, P. S.; Szychowski, B.; Dockery, L. T.; Pak, Y. J.; Li, W.; Kane, M. A.; Swaan, P.; Daniel, M.-C. Set of Highly Stable Amine- and Carboxylate-Terminated Dendronized Au Nanoparticles with Dense Coating and Nontoxic Mixed-Dendronized Form. *Langmuir* **2019**, *35* (9), 3391-3403, 10.1021/acs.langmuir.8b03196. DOI: 10.1021/acs.langmuir.8b03196.
